# Diterpenes Synthesized from the Natural Serrulatane Leubethanol and Their *in Vitro* Activities against *Mycobacterium tuberculosis*

**DOI:** 10.3390/molecules20047245

**Published:** 2015-04-21

**Authors:** Ricardo Escarcena, Jonathan Perez-Meseguer, Esther del Olmo, Blanca Alanis-Garza, Elvira Garza-González, Ricardo Salazar-Aranda, Noemí Waksman de Torres

**Affiliations:** 1 Department of Pharmaceutical Chemistry, Faculty of Pharmacy, CIETUS, IBSAL, Universidad de Salamanca, 37007-Salamanca, Spain; E-Mail: ricar@usal.es; 2 Department of Analytical Chemistry, Faculty of Medicine, Universidad Autónoma de Nuevo Leon, PO Box 2316, Sucursal Tecnológico, Monterrey, NL 64841, Mexico; E-Mails: jonathanmeseguer@hotmail.com (J.P.-M.); alanisaliciab@yahoo.com.mx (B.A.-G.); salazar121212@yahoo.com.mx (R.S.-A.); nwaksman@gmail.com (N.W.T.); 3 Servicio de Gastroenterología y Departamento de Patología Clínica, Hospital Universitario Dr. José Eleuterio González, Universidad Autónoma de Nuevo Leon, Av. Madero y Gonzalitos S/N, Mitras Centro, Monterrey, NL 64220, Mexico; E-Mail: elvira_garza_gzz@yahoo.com

**Keywords:** serrulatane derivatives, leubethanol, semi-synthesis, *in vitro* antimycobacterial activity, cytotoxicity assay

## Abstract

Seventeen new derivatives of the natural diterpene leubethanol, including some potential pro-drugs, with changes in the functionality of the aliphatic chain or modifications of aromatic ring and the phenolic group, were synthesized and tested *in vitro* by the MABA technique for their activity against the H37Rv strain of *Mycobacterium tuberculosis*. Some compounds showed antimycobacterial selectivity indices higher than leubethanol.

## 1. Introduction

Tuberculosis (TB) caused by *Mycobacterium tuberculosis* (*Mtb*) remains a worldwide health problem. According to the latest report from the World Health Organization (WHO), the number of people suffering from TB in 2013 was 9.0 million and approximately 1.5 million people died from the disease [1]. Moreover, *Mtb* is highly infectious; it is reported that about one third of the world’s population is latently infected, and that 10% of this population will develop active disease. A further significant problem is the association with HIV infection, from 1.5 million deaths caused by TB in 2013, 0.36 million deaths were in HIV positive people. Therefore, TB-HIV co-infection is a major public health problem [1]. 

The current first line drugs for TB treatment (isoniazid (INH), rifampicin (RIF), pyrazinamide and ethambutol) were discovered decades ago and are increasingly becoming less useful due to emerging multidrug-resistant (MDR), extended (XDR) or extremely (XXDR) resistant strains [2]. There were an estimate of 480,000 new cases of multidrug-resistant tuberculosis (MDR-TB) and 170,000 MDR-TB-related deaths. Treatment of MDR-TB disease requires high resources and at least the administration of RIF and INH in combination with second-line drugs. Second line drugs are more expensive, more toxic, and less effective than drugs used in standard therapy [3]. These facts have motivated the search for new drugs and treatment strategies. New drug candidates should shorten conventional chemotherapy and be effective against MDR-TB.

Natural products have played an important role in the discovery of new drugs, as is the chemotherapy of tuberculosis [4,5], with the discovery of streptomycin, capreomycin, cycloserine, or development of rifamycin derivatives. Now, there is a re-emerging interest in natural products as novel template for the development of new drugs and particularly suitable as antibacterial leads [6,7,8]. Compounds bearing a serrulatane skeleton have been reported as good antimycobacterial agents [9,10,11]. 

**Figure 1:**
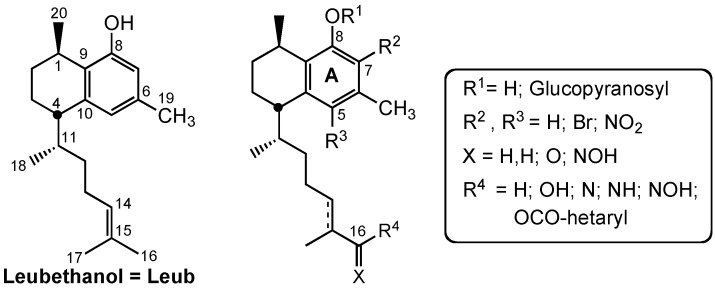
Leubethanol modifications on ring A and the alkyl side chain.

Waksman *et al.* isolated leubethanol (Leub), a natural serrulatane diterpenoid, from *Leucophyllum frutescens* methanolic extracts [10] and introduced different modifications on the skeleton to improve antimycobacterial activity [12]. Taking into account previous results of the research group on leubethanol structure modification related to antimycobacterial activity [12] and the information of similar structures reported in the literature [9,11], we intended to explore in the present study new possibilities to obtain better antimycobacterial compounds by introducing a glycosylated fragment on Leub phenolic hydroxyl, substitutions on the aromatic ring A, and new side-chain functionalizations (Figure 1).

## 2. Results and Discussion

### 2.1. Chemistry 

Leubethanol 8-*O*-β-d-glucopyranosyl derivative **1** was obtained with Schmidt trichloroacetimidate as glucosyl donor. Thus, Leub was treated with 2,3,4,6-tetra-*O*-acetyl*-*α*-*d-glucopyranosyl trichloroacetimidate [13] and BF_3_·OEt as Lewis acid catalyst in dichloromethane [14] to give the 8-*O*-(2,3,4,6-tetra-*O*-acetyl-β-d-glucopyranosyl)leubethanol as the only adduct in 80% yield. When acetonitrile was used as reaction solvent instead of dichloromethane the reaction did not proceed [15]. In previous attempts to obtain the tetra-*O*-acetyl-glucopyranosyl Leub derivative by using 2,3,4,6-tetra-*O*-acetyl-α-d-glucopyranosyl bromide with potassium carbonate in chloroform [16], silver carbonate in pyridine [17], or transfer-phase catalysis with tetrabutylammonium bromide [18] no glycosidation product was obtained.

Inversion of the anomeric carbon configuration of the 8-*O-*β*-*d-glucopyranosyl Leub derivative was established by a study of the H-1' coupling constant, with a value of 3.7 Hz in the glucopyranosyl trichloroacetimidate and of 7.9 Hz in the Leub aduct. Afterwards, the obtained Leub derivative was treated with sodium ethoxide in ethanol to provide compound **1** in 52% yield. Unequivocal structural assignment was achieved by 2D-NMR experiments (^1^H,^1^H-COSY, HMQC, HMBC).

**Scheme 1:**
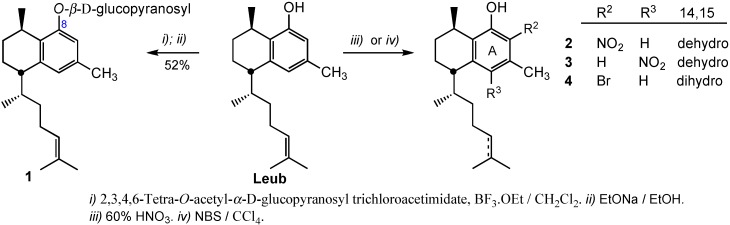
Modifications of leubethanol on the phenolic hydroxyl and aromatic ring A.

We introduced electron withdrawing groups (NO_2_, Br) on ring A. Leub treatment with 65% HNO_3_ gave a mixture of 7-nitro and 5-nitro derivatives, (compounds **2** and **3**, respectively) in 1.5:1.0 ratio. The unambiguous structural assignment of the nitrated derivatives was established by NOE experiments. Irradiation of the proton at 6.50 ppm on compound **2** showed a NOE effect on two of the methyls, the aromatic methyl at 2.21 ppm and the methyl doublet on the aliphatic chain at 0.83 ppm. On the other hand, irradiation of the proton at 6.63 ppm on compound **3** showed a NOE effect on the hydroxylic proton at 11.27 ppm and on the aromatic methyl at 2.57 ppm. The aliphatic double bond of Leub was hydrogenated with H_2_ in the presence of Pd-C catalyst, and the resulting 14,15-dihydroleubethanol treated with *N*-bromosuccinimide (NBS) to provide 7-bromoleubethanol (**4**) in 67% yield (Scheme 1). As just mentioned in the nitro derivatives, the bromine atom introduction at Leub position 7 was unequivocally established by NOE experiments.

**Scheme 2:**
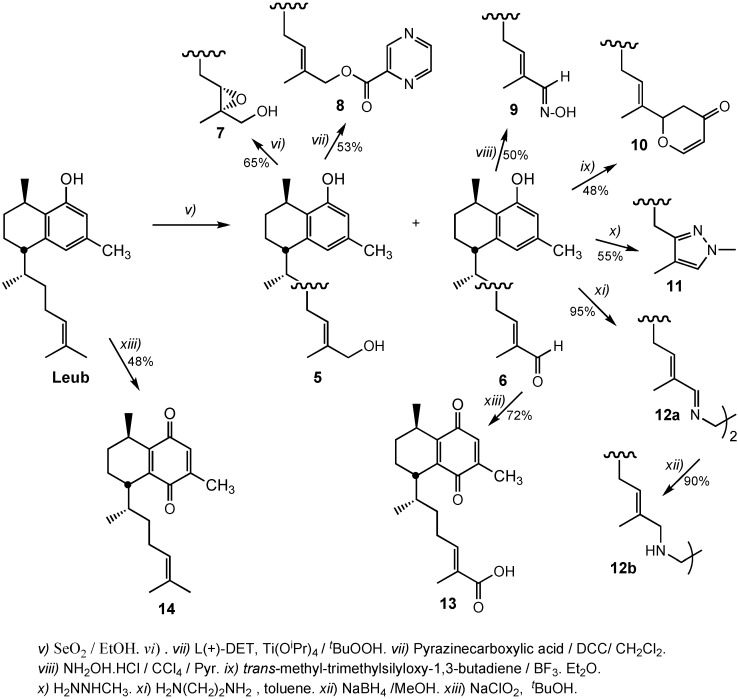
Synthesis of leubethanol derivatives modified at the side chain.

Then, some modifications on the side chain were performed (Scheme 2). First, we obtained the allylic alcohol **5** and the α,β-unsaturated aldehyde **6** (in 2.2:1 proportion) by Leub treatment with selenium dioxide. Treatment of compound **5** under Sharpless asymmetric epoxidation conditions (with l-(+)diethytartrate) gave the corresponding α-oxirane derivative **7** in 65% yield and ~91% ee. The allylic alcohol **5** was condensed with pyrazinecarboxylic acid in the presence of *N,N*-dicyclohexyl-carbodiimide (DCC) as coupling reagent to give the pyrazine ester derivative **8** in 53% yield. Compound **8** showed ^1^H-NMR signals at 9.29 ppm (1H, d, *J* = 1.6 Hz), 8.75 ppm (1H, d, *J* = 2.4 Hz) and 8.73 (1H, dd, *J* = 2.4; 1.6 Hz) of the pyrazine ring. Attempts to obtain other esters by treatment with 2-amino-isonicotinic acid or with 6-aminopyridine-3-carboxylic acid reaction did not progress, even by changing the coupling reagent (1-ethyl-3-(3-dimethylaminopropyl)carbodiimide, EDCI), the reaction solvent, or by increasing reaction time or the temperature. 

Furthermore, the α,β-unsaturated aldehyde **6** was used to obtain compounds **9**–**13**. Treatment of compound **6** with hydroxylamine hydrochloride in dichloromethane/pyridine provided the hydroxyimine **9**, which showed two singlet signals at 7.66 and 8.09 ppm in its ^1^H-NMR for the hydroxyimine group. The dihydropyranone **10** was obtained by reaction of **6** with *trans*-methoxy-trimethylsilyloxy-1,3-butadiene in the presence of boron trifluoride diethyl ether with 48% yield [19]. Compound **10** was completely characterized by 2D-NMR experiments (^1^H, ^1^H-COSY, HMQC, HMBC). The dihydropyranone fragment showed the ^1^H-NMR signals at 4.69 ppm (1H, dd, *J* = 14.5, 2.4 Hz, H-16; 84.5 ppm in the ^13^C-NMR), 2.73 ppm (1H, dd, *J* = 16.7, 14.5 Hz) and 2.35 ppm (1H, m) of the methylene (H-24) and at 7.39 (1H, d, *J* = 7.1 Hz, H-21) and 5.52 ppm (1H, d, *J* = 7.1 Hz, H-22) of the olefinic protons. Treatment of **6** with methylhydrazine in 2-propanol under refluxing conditions gave the pyrazole **11** with 55% yield [20]. Compound **11** showed in its ^1^H-NMR a one olefinic proton singlet at 7.00 ppm (H-16) and a methyl at 3.75 ppm of the CH_3_-N. Two moles of **6** were treated with one mole of ethylenediamine in anhydrous toluene and a large excess of anhydrous sodium sulfate at room temperature to give a diimine **12a**, which was reacted with sodium borohydride in ethanol to provide the ethylenediamine **12b**, in a total yield of 86%. Finally, compound **6** was treated with sodium chlorite [21] to oxidize the aldehyde to an acid. The additional oxidation on the aromatic ring was observed. Compound **13** showed a quinone fragment with chemical shifts at 188.4 and 187.1 ppm of carbons C-5 and C-8, respectively, in addition to a new carbonyl at 173.00 ppm of the acid group. Only one aromatic proton was appreciated in ^1^H-NMR at 6.52 ppm. To compare the influence of the quinone fragment on the antimycobacterial activity, we decided to obtain the quinone of Leub, compound **14**.

**Scheme 3:**
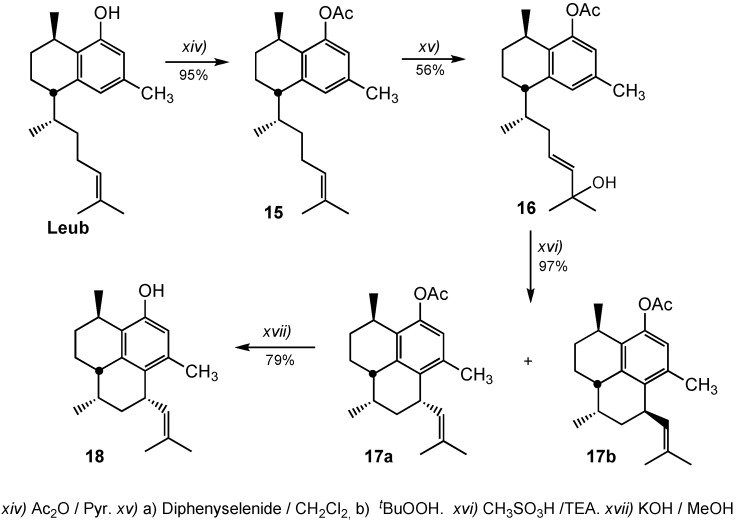
Synthesis of the tricyclic derivative **18** from leubethanol.

Promising antimycobacterial activity against *Mtb* H37Rv strain has been reported in the literature [9] for pseudopteroxazole and its analogue *seco*-pseudopteroxazole (MIC values of 97% and 66% mycocacterial growth inhibition at 12.5 μg/mL). Then, we decided to obtain the tricyclic diterpene analogue of Leub (Scheme 3).

The phenol group was protected as an acetate by reaction of Leub with acetate anhydride in the presence of pyridine. Compound **15** showed a methyl group at 2.12 ppm in ^1^H-NMR and two acetyl group signals at 169.7 and 21.2 ppm in the ^13^C-NMR. Then, the allylic alcohol **16** was obtained in 56% yield by compound **15** treatment with phenylseleninic acid (obtained *in situ* from diphenyl deselenide and hydrogen peroxide) followed by *tert*-butyl-hydroperoxide. Compound **16** displayed two singlet methyl groups at 1.22 and 1.23 ppm, and two olefinic protons at 5.30 ppm as a multiplet, and at 5.51 ppm as a doublet (*J* = 15.8 Hz) in the ^1^H-NMR. Cyclization of compound **16** was achieved using methanesulfonic acid in CH_2_Cl_2_ at −40 °C. Although the cyclization proceeded quantitatively, the product was a mixture of diastereomers **17a** and **17b** in a ratio of about 2:1. Compound **17a** could be separated by silica gel chromatography, while compound **17b** was obtained as a mixture of **17a** and **17b**. In the case of **17a**, the vinyl proton attached to C(14) appeared at δ 4.99 as a broad doublet, *J =* 9.2 Hz while in **17b** it was observed at δ 5.14 (doublet, *J =* 9.2 Hz). Compound **17a** saponification with KOH/MeOH finally gave the phenol derivative **18**. The overall yield from Leub to **18** was 41%.

### 2.2. Antimycobacterial Activity 

The anti-MTB activity was assessed *in vitro* against the H37Rv strain (ATTC 27294) [22] susceptible to all SIREP anti-TB drugs, according to a modified Microplate Alamar Blue Assay (MABA) [23]. Ethambutol (EMB) was used as the reference drug and the assays were performed by triplicate independent experiments. Cytotoxicity on Vero cells [24] was also measured for those compounds with an appreciable *in vitro* anti-MTB effect.

The *in vitro* antimycobacterial results of the Leub and seventten derivatives are shown in Table 1. The introduction of a glycoside fragment on the phenolic group induces the loss of the activity. We knew that the introduction of acyl or alkyl fragments at this position were not convenient for the activity, but we expected that the introduction of other hydroxyl groups (as is a glycoside) would not modify the activity results, so this indicates that phenol acidic properties are crucial for good activity. Substitutions at position 5 or 7 on Leub were also relevant for the antimycobacterial activity. We had previously found that the introduction of aromatic substituents on position 5 maintained Leub activity [12]. Taken into account the antimycobacterial activity results found for the pseudopterosins [9], compounds with an oxazole between positions 7 and 8 of the serrulatane skeleton, we expected to see good activity results for compounds substituted at position 7, like compounds **2** and **4**, but they did not show activity. The antimycobacterial activity of compound **3** was a slightly lower than that of Leub, with small increase in the selectivity index. We planned to obtain the bromine derivative at position 5 to compare the antimycobacterial activity with compound **3**, for this we had to previously protect the phenol group with a bulky group to force the introduction of the bromine at position 5, but in the process of removing the protecting group we got a mixture of compounds of difficult separation. As we indicated in the previous work functionalization of the C-16 methyl, compounds **5** and **6** showed similar potency and selectivity index to the natural compound leubethanol, 

Modifications of the aliphatic chain by introduction of an oxirane on the 14,15-double bond, (compound **7**), or modifications on the alcohol or aldehyde functions, pyrazine ester **8**, the hydroxyimine **9**, or the heterocyclic compounds **10** and **11** led to the loss of activity. Only Leub dimer **12a**, tricyclic derivative **18** and especially the ethylenediamine **12b** maintained the antimycobacterial activity. Regarding the Vero cells/*Mtb* selectivity, compounds **3**, **5**, **6** and **18** showed similar selectivity to leubethanol, and compound **12b** twice as much as leubethanol. 

**Table 1:** Antimycobacterial activity, cytotoxicity and selectivity index of leubethanol derivatives. 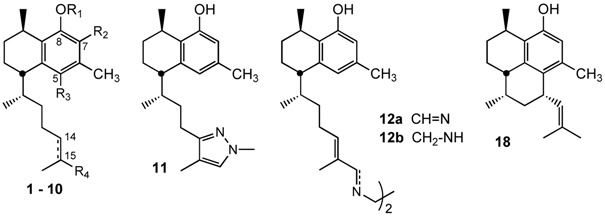

Comp.	14,15-	R_1_	R_2_	R_3_	R_4_	*MTB* H_37_Rv MIC (μM)	Vero Cells CC_50_ (μM)	Selectivity Index (SI)
**Leub**	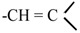	H	H	H	CH_3_	**25.1**	**123**	**4.9**
**1**	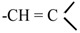	Gluc	H	H	H	>200	nd	--
**2**	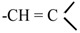	H	NO_2_	H	CH_3_	>200	nd	--
**3**	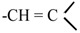	H	H	NO_2_	CH_3_	**47.2**	**251**	**5.3**
**4**	-CH_2_-CH-	H	Br	H	CH_3_	>200	nd	--
**5**	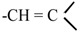	H	H	H	CH_2_OH	**25.8**	**132**	**5.1**
**6**	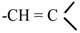	H	H	H	CHO	**25.8**	**116**	**4.5**
**7**	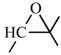	H	H	H	CH_2_OH	>200	nd	--
**8**	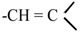	H	H	H	CH_2_OCOC_4_H_3_N_2_	>200	nd	--
**9**	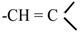	H	H	H	C=N-OH	>200	nd	--
**10**	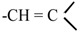	H	H	H	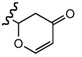	>200	nd	--
**11**						>200	nd	--
**12a**						**100.1**	nd	--
**12b**						**24.8**	**215**	**8.7**
**13**						>200	nd	--
**14**						>200	nd	--
**18**						**55.1**	**173**	**3.1**
**EMB**						**7.5**	**>500**	**>67**

MIC and CC_50_: rounded media values of three experiments; nd = no determined; --: not calculated; Selectivity index calculated by the equation: SI = CC_50_ (Vero)/MIC(H_37_Rv). EMB = Ethambutol. Gluc = β-d-glucopyranosyl.

## 3. Experimental Section 

### 3.1. General Information 

All commercial chemicals and solvents used were reagent grade. Flash column chromatography was done using Merck Silica Gel 60 (0.04–0.063 mm). Reactions were monitored by TLC using Merck 60F_254_ silica gel plates. Compounds were detected visually under UV irradiation (254 nm) and by spraying with sulfuric acid and phosphomolybdic acid reagents followed by heating at 100 °C. ^1^H-NMR and ^13^C-NMR spectra were obtained with a Bruker AC 200 spectrometer (200 and 50.3 MHz, respectively). Chemical shifts were recorded in parts per million (ppm, δ) and were reported relative to the solvent peak or TMS. High resolution mass spectra (HRMS) were measured with a QSTAR XL quadrupole time-of-flight mass spectrometer, by direct injection on the sample dissolved in MeOH and Ionization voltage of 5500 V. Infrared (IR) spectra were measured on a Nicolet Impact 410 spectrophotometer. The absorbances were measured with a Bio-Rad Benchmark model microplate reader. Middlebrook 7H9 broth medium was obtained from Difco^®^ (Becton Dickinson and Co., Sparks, MD, USA) resazurin from Probiotek^®^ (Hayward, CA, USA), and 0.45 μm pore size, 13 mm diameter PTFE from Millipore Millex^®^ (Millipore Co., Bedford, MA, USA) Isoniazid, rifampicin, penicillin, streptomycin, and MTT were obtained from Sigma^®^ (St. Louis, MO, USA) and fetal bovine serum from Hyclone^®^ (Logan, UT, USA).

### 3.2. Chemistry 

#### 3.2.1. Preparation of 8-*O*-(2,3,4,6-Tetra-*O*-acetyl-β-d-glucopyranosyl)leubethanol

To a stirred solution of leubethanol (112 mg, 0.40 mmol) in dry CH_2_Cl_2_ (5 mL) at −20 °C, BF_3_·Et_2_O (75 μL, 0.60 mmol) with a syringe was added. Immediately, the suspension turned into a light-yellow solution. The mixture was stirred for 45 min and then, a solution of 2,3,4,6-tetra-*O*-acetyl-α-d-glucopyranosyl trichloroacetimidate (292 mg, 0.59 mmol), recently obtained, in CH_2_Cl_2_ (2 mL) was added. The reaction was maintained for 4 h at room temperature. After that, the solution was diluted with CH_2_Cl_2_ and was washed with 10% NaHCO_3_, brine, water and dried over sodium sulphate. The organic layer was concentrated to give an oil, that was purified by column chromatography with *n*-hexane/ethyl acetate (95:5) as eluent to give 207 mg (80%) of 8-*O*-(2,3,4,6-tetra-*O*-acetyl-β-d-glucopyranosyl) leubethanol. IR: 2956, 2928, 2870, 1755, 1696, 1612, 1606, 1575, 1516, 1450, 1378, 1226, 1108, 1043, 832, 750 cm^−1^; ^1^H-NMR (CDCl_3_): δ 0.95 (3H, d, *J* = 6.8 Hz), 1.11 (3H, d, *J* = 5.7 Hz), 1.1–1.3 (2H, m), 1.54 (3H, s), 1.65, (3H, s), 1.16–2.0 (7H, m), 2.03 (3H, s), 2.04 (3H, s), 2.05 (3H, s), 2.09 (3H, s), 2.26 (3H, s), 2.53 (1H, m), 2.88 (1H, m), 3.89 (1H, m), 4.21 (2H, m), 5.00 (1H, d, *J* = 6.8), 5.15 (1H, t, *J* = 8.8 Hz), 5.33 (3H, m), 6.71 (1H, s), 6.72 (1H, s); ^13^C-NMR (CDCl_3_): δ 17.7, 18.8, 19.3, 20.7, 21.6, 22.7 (4C), 25.8, 26.2, 26.8, 27.5, 33.2, 42.4, 37.7, 62.4, 68.6, 71.2, 71.9, 72.9, 99.3, 113.8, 124.9 (2C), 129.9, 131.1, 134.8, 140.9, 154.6; 169.3, 169.6, 170.4, 170.7; HRMS (ESI^+^) for C_34_H_48_O_10_Na [M+Na]^+^ calcd. 639.3145; found 639.3110.

#### 3.2.2. Preparation of 8-*O*-(β-d-Glucopyranosyl)leubethanol (**1**)

2,3,4,6-Tetra-*O*-acetyl-β-d-glucopyranosyl leubethanol (160 mg, 0.25 mmol) was dissolved in dry EtOH (5 mL) and a solution of sodium ethoxide (28 mg Na, 1.25 mmol) in EtOH was added. After stirring the mixture at room temperature for 2 h the reaction was completed. The solution was taken to dryness to obtain a residue that was purified by column chromatography with CH_2_Cl_2_/MeOH (8:2) as eluent to give 58 mg (52%) of compound **1**. IR: 3412, 2923, 2862, 1652, 1612, 1575, 1458, 1376, 1268, 1075, 841 cm^−1^; ^1^H-NMR (400 MHz, CDCl_3_): δ 0.95 (3H, d, *J* = 6.7 Hz), 0.96–1.30 (4H, m), 1.30 (3H, d, *J* = 6.8 Hz), 1.51 (3H, s), 1.60–2.0 (8H,m), 1.62 (3H, s), 2.24 (3H, s), 2.57 (1H, m), 3.28 (1H, m), 3.32 (1H, m), 3.41 (1H, m), 3.49 (1H, m), 3.70 (1H, dd, *J* = 12.1, 5.8), 3.89 (1H, dd, *J* = 12.1, 2.0), 4.87 (1H, d, *J* = 7.3 Hz), 4.93 (1H, t, *J* = 6.8 Hz), 6.63 (1H, s), 6.79 (1H, s); ^13^C-NMR (50.3 MHz, CDCl_3_): δ 17.7, 19.1, 20.3, 21.6, 22.2, 25.9, 27.4, 27.5, 28.6, 34.6, 39.5, 43.7, 62.7, 71.6, 78.1, 78.4, 75.2, 102.2, 113.7, 124.6, 125.9, 132.5, 132.0, 136.0, 141.2, 156.6; HRMS (ESI^+^) for C_26_H_41_O_6_ [M+H]^+^ calcd. 449.2903; found 449.2937.

#### 3.2.3. Preparation of 7*-*Nitroleubethanol (**2**) and 5-Nitroleubethanol (**3**)

To a solution of leubethanol (56 mg, 0.19 mmol) in *n*-hexane (2 mL) 65% HNO_3_ (16 μL, 0.23 mmol) was added and the mixture stirred for 8 h. Then, ethyl acetate (30 mL) was added and the organic layer was washed with 5% NaHCO_3_, water and dried over Na_2_SO_4_. The solvent was removed under vacuum to provide a crude (60 mg), that was purified by silica flash chromatography using *n*-hexane/ethyl acetate (95:5) as eluent, to give 23 mg (37%) of 7-nitroleubethanol (**2**) and 16 mg (26%) of 5-nitroleubethanol (**3**).

*Compound* **2**. IR: 3450, 2959, 2927, 2870, 1606, 1575, 1516, 1453, 1352, 1239, 1181, 1128, 1052 cm^−1^; ^1^H-NMR (CDCl_3_): δ 0.83 (3H, d, *J* = 6.8 Hz), 1.18 (3H, d, *J* = 6.8 Hz), 1.3–1.1 (2H, m), 1.52 (3H, s), 1.65 (3H, s), 2.0–1.6 (7H, m), 2.21 (3H, s), 3.12 (2H, m), 4.93 (1H, t, *J* = 6.8 Hz), 5.37 (1H, br s), 6.50 (1H, s); ^13^C-NMR (CDCl_3_): δ 17.6, 18.2, 18.5, 19.0, 21.5, 25.4, 25.7, 26.6, 26.7, 34.7, 37.7, 38.0, 114.7, 124.5, 128.4, 129.0, 131.6, 134.0, 146.3, 154.1; HRMS (ESI^+^) for C_20_H_29_NO_3_Na [M+Na]^+^ calcd. 354.2045; found 354.2042.

*Compound* **3**. IR: 2959, 2928, 2871, 1601, 1577, 1540, 1454, 1406, 1339, 1272, 1201, 1109, 1056 cm^−1^. ^1^H-NMR (CDCl_3_) δ 0.99 (3H, d, *J* = 6.8 Hz), 1.19 (3H, d, *J* = 6.8 Hz), 1.3–1.1 (2H, m), 1.54 (3H, s), 1.65 (3H, s), 2.0–1.6 (7H, m), 2.57 (3H, s), 3.24 (1H, m), 4.95 (1H, t, *J* = 6.8 Hz), 6.63 (1H, s), 11.27 (1H, br s); ^13^C-NMR (CDCl_3_): δ 17.7, 18.8, 18.8, 22.9, 22.9, 25.8, 26.2, 26.9, 27.0, 33.4, 37.9, 42.9, 124.5, 124.7, 131.5, 131.6, 132.2, 132.6, 140.9, 154.1; HRMS (ESI^+^) for C_20_H_29_NO_3_Na [M+Na]^+^ calcd. 354.2045; found 354.2033.

#### 3.2.4. Preparation of 7-Bromo-14,15-dihydroleubethanol (**4**)

A solution of leubethanol (306 mg, 1.1 mmol) in ethanol (10 mL) was treated with H_2_ under 10% Pd-C catalysis to provide 14,15-dihydroleubethanol in 95% yield [12]. Then, 14,15-dihydroleubethanol (54 mg, 0.18 mmol) was dissolved in carbon tetrachloride (3 mL) and NBS (35 mg, 0.20 mmol) was added. The mixture was refluxed for 4 h, followed to 1 h at 0 °C. The solid residue was filtered off and the solvent was evaporated under reduced pressure to give a crude oil that was purified by silica flash chromatography using *n*-hexane/ethyl acetate (98:2) as eluent giving 44 mg (67%) of compound **4**. IR: 3511, 2952, 2927, 2869, 1599, 1559, 1459, 1399, 1379, 1312, 1235, 1207, 1171, 1040, 980 cm^−1^. ^1^H-NMR (CDCl_3_): δ 0.83 (6H, d, *J* = 6.8 Hz), 0.94 (3H, d, *J* = 6.8 Hz), 1.10–1.30 (2H, m), 1.19 (3H, d, *J* = 7.2 Hz), 1.50–2.0 (8H, m), 2.34 (3H, s), 2.54 (1H, m), 3.18 (1H, m), 5.65 (1H, br s), 6.66 (1H, s); ^13^C-NMR (CDCl_3_): δ (ppm) 18.9, 19.4, 21.0, 22.6, 22.8, 23.0, 25.6, 27.2, 27.5, 28.0, 33.6, 38.5, 39.2, 42.2, 110.4, 123.3, 128.2, 133.8, 139.9, 149.3; HRMS (ESI^+^) for C_20_H_32_BrO [M+H]^+^: calcd 367.1637; found 367.1646.

#### 3.2.5. Preparation of 14,15-α-Epoxy-16-hydroxyleubethanol (**7**)

l-(+)DET (60 μL, 0.34 mmol) was dissolved in CH_2_Cl_2_ (3.5 mL) under nitrogen and the solution cooled to −23 °C, then Ti(*i*-PrO)_4_ (300 μL, 6M) was added and the mixture stirred for 15 min. After that, a solution of compound **5** (25 mg, 0.08 mmol) in CH_2_Cl_2_ (1 mL) [12], was added and the mixture stirred for 5 min. Then, a solution of 3M of *tert*-butyl hydroperoxide (TBHP) in toluene (0.2 mL, 0.6 mmol) was added and the mixture stirred for 12 h. The reaction was quenched by the addition of a 10% aq. solution of tartaric acid (1 mL) and was stirred for 30 min at −23 °C, followed of 1 h at room temperature. The solution was filtered off through Celite, and the residue washed with diethyl ether. The organic layer was washed with water and reduced to a volume of 50 mL, treated with 1 M solution of NaOH (3 mL) at 0 °C for 45 min. The organic phase was washed with brine. The aqueous layer was extracted with Et_2_O and washed with brine. The combined organic layers were dried over sodium sulphate and concentrated to give an oil, which was purified by column chromatography with *n*-hexane/ethyl acetate (7:3) as eluent to give 13.6 mg (65%) of 14,15-α-epoxy-16-hydroxy-leubethanol (**7**) in ≈91% ee. IR: 3408, 2951, 2927, 2869, 1616, 1580, 1457, 1421, 1377, 1332, 1285, 1254, 1035, 842 cm^−1^; ^1^H-NMR (CDCl_3_): δ 0.98 (3H, d, *J* = 6.8 Hz), 1.20 (3H, d, *J* = 6.8 Hz), 1.21 (3H, s), 2.24 (3H, s), 2.60 (1H, m), 2.93 (1H, m), 3.07 (1H, m), 3.63 (2H, m), 4.77 (1H, br s), 6.43 (1H, s), 6.57 (1H, s); ^13^C-NMR (CDCl_3_): δ 14.2, 18.8, 21.1 (2C), 26.6, 26.6, 27.4, 30.0, 38.5, 39.2, 42.3, 60.4 (2C), 65.3, 114.4, 122.2, 126.3, 135.3, 140.7, 153.1; HRMS (ESI) for C_20_H_31_O_2_ [M+H]^+^: calcd 303.2324; found 303.2369.

#### 3.2.6. Preparation of (*E*)-6*R*-((1*S*,4*R*)-5-Hydroxy-4,7-dimethyl-1,2,3,4-tetrahydronaphthalen-1-yl)-2-methylhept-2-enyl pyrazine-2-carboxylate (**8**)

A solution of pyrazinecarboxylic acid (13.6 mg, 0.11mmol), allylic alcohol **5** (31.5 mg. 0.1 mmol), and *N,N*-dicyclohexylcarbodiimide (22.7 mg, 0.11 mmol) in 5 mL of dichloromethane was stirred for 24 h at room temperature. The mixture was filtered off and the filtrate washed with water, 5% HCl, and with water again, dried over sodium sulphate and the solvent removed under vacuum to give a crude oil that was purified by silica flash chromatography using *n*-hexane/ethyl acetate (9:1) as eluent to give 10 mg (33%) of starting material and 22 mg (53%) of pyrazine ester derivative **8**. IR: 3452, 2949, 2919, 1702, 1616, 1580, 1453, 1332, 1247, 1001, 890 cm^−1^; ^1^H-NMR (CDCl_3_); δ 0.96 (3H, d, *J* = 6.8 Hz), 1.1–1.6 (6H, m), 1.18 (3H, d, *J* = 7.2 Hz), 1.68 (3H, s), 1.9–2.1 (6H, m), 2.21 (3H, s), 2.56 (1H, m), 3.05 (1H, m), 4.78 (2H, s), 5.47 (1H, t, *J* = 7.2 Hz), 6.43 (1H, s), 6.55 (1H, s), 8.73 (1H, dd, *J* = 1.6, 2,4 Hz); 8.75 (1H, d, *J* = 2,4 Hz), 9.29 (1H, d, *J* = 1.6 Hz); ^13^C-NMR (CDCl_3_): δ 14.1, 18.7, 19.3, 21,1, 21.1, 26.2, 26.6, 27.4, 32.8, 38.2, 42.3, 72.2, 113.3, 122.3, 126.3.128.9, 131.8, 135.1, 140.7, 143.6, 144.5, 146.2, 147.5, 153.2, 163.8. HRMS (ESI^+^) for C_25_H_32_N_2_O_3_Na [M+Na]^+^: calcd 431.2311; found 431.2304.

#### 3.2.7. Preparation of 16-Hydroximinoleubethanol (**9**)

The 16-oxoleubethanol **6** (43 mg, 0.14 mmol) dissolved in methanol (5 mL) was treated with hydroxylamine hydrochloride (97 mg, 1.4 mmol) and 5 drops of pyridine and the mixture was refluxed for 14 h. The solvent was removed under vacuum and the crude dissolved in dichloromethane, the organic layer was washed with water and dried over Na_2_SO_4_. Removal of the solvent gave 40 mg of a crude, that was purified by silica flash chromatography using *n*-hexane/ethyl acetate (9:1) as eluent, to provide 20 mg (50%) of **9**. IR: 3367, 2922, 2862, 1616, 1580, 1454, 1372, 1321, 1285, 1253, 1171, 1054, 995 cm^−1^; ^1^H-NMR (CDCl_3_): δ 0.99 (3H, d, *J* = 6.8 Hz), 1.3–1.6 (4H, m), 1.20 (3H, d, *J* = 6.8 Hz), 1.77 (3H, s), 1.8–2.2(6H, m), 2.24 (3H, s), 2.60 (1H, m), 3.06 (1H, m), 5.59 (1H, t, *J* = 7.6 Hz), 6.43 (1H, s), 6.56 (1H, s), 7.66 (1H, s), 8.10 (1H, br s); ^13^C-NMR (CDCl_3_): δ 11.9, 18.6, 19.2, 21.0, 21.4, 26.5, 26.5, 27.5, 32.5, 38.2, 42.2, 113.3, 122.3, 126.2, 130.2, 135.2, 139.9, 140.6, 153.0, 154.9; HRMS (ESI+) for C_20_H_30_NO_2_ [M+H]^+^ calcd. 316. 2277; found 316.2271.

#### 3.2.8. Preparation of 2-((*E*)-6*R*-((1*S*,4*R*)-5-Hydroxy-4,7-dimethyl-1,2,3,4-tetrahydronaphthalen-1-yl)hept-2-en-2-yl)-2*H*-pyran-4(3*H*)-one (**10**)

To a solution of *trans*-methoxytrimethylsilyloxy-1,3-butadiene (40 mg, 0.23 mmol) in dry diethyl ether (5 mL) under nitrogen atmosphere at −30 °C aldehyde **6** (75 mg, 0.23 mmol) dissolved in diethyl ether (2 mL) and boron trifluoride diethyl etherate (45 μL, 0.23 mmol) were added. The mixture was maintained for 4 hours under stirring and the reaction was quenched by the addition of Et_3_N (96 μL, 0.69 mmol) and water (2 mL). The mixture was treated with diethyl ether, washed with brine and the organic layer dried with Na_2_SO_4_. The crude was dissolved in dichloromethane (5 mL) and 4 drops of trifluoroacetic acid was added. One hour later, the solvent was evaporated under reduced pressure to give a crude that was purified by silica flash chromatography using *n*-hexane/ethyl acetate (9:1) as eluent giving 20 mg (26%) of starting material and 38 mg (48%) of dihydropyranone derivative **10**. IR: 3402, 2953, 2923, 2869, 1661, 1588, 1502, 1454, 1413, 1278, 1226, 1174, 1050, 984 cm^−1^; ^1^H-NMR (CDCl_3_): δ 0.99 (3H, d, *J* = 6.8 Hz), 1.19 (3H, d, *J* = 6.9 Hz), 2.23 (3H, s), 2.35 (3H, s), 2.59 (1H, m), 2.73 (1H, dd, *J* = 16.7, 14.5 Hz), 3.08 (1H, m). 4.69 (1H, dd, *J* = 14.5, 2.4 Hz), 5.42 (2H, m), 6.44 (1H, s), 6.56 (1H, s), 7.39 (1H, dt, *J* = 6.1, 1.0 Hz); ^13^C-NMR (CDCl_3_): δ 11.9, 18.7, 19.2, 21.1, 21.1, 26.0, 26.5, 27.5, 32.7, 38.2, 40.6, 42.2, 84.5, 106.7, 113.3, 122.2, 126.3, 130.8, 131.2, 135.0, 140.6, 153.2, 163.6, 193.2; HRMS (ESI^+^) for C_24_H_32_O_3_Na [M+Na]^+^ calcd. 391.2249; found 391.2239.

#### 3.2.9. Preparation of (5*S*,8*R*)-5-((2*R*)-4-(1,4-Dimethyl-1*H*-pyrazol-3-yl)butan-2-yl)-3,8-dimethyl-5,6,7,8-tetrahydronaphthalen-1-ol (**11**)

To a stirring solution of 16-oxoleubethanol **6** (19 mg, 0.06 mmol) in 2-propanol (2 mL), methylhydrazine (26 μL, 0.5 mmol) was added and the mixture refluxed for 5 hours. The solvent was removed under reduced pressure to give a crude oil that was purified by silica flash chromatography using *n*-hexane/ethyl acetate (9:1) as eluent to give 11 mg (55%) of the pyrazole **11**. IR: 3209, 2929, 2864, 1613, 1578, 1451, 1419, 1371, 1321, 1285, 1260, 1172, 1059 cm^−1^. ^1^H-NMR (CDCl_3_): δ 1.02 (3H, d, *J* = 6.8 Hz), 1.10–1.30 (2H, m), 1.18 (3H, d, *J* = 7.2 Hz), 1.50–203 (8H, m), 1.85 (3H, s), 2.20 (3H, s), 2.59 (1H, m), 3.02 (1H, m), 3.75 (3H, s), 5.31 (1H, br s), 6.40 (1H, s), 6.50 (1H, br s), 7.00 (1H, br s); ^13^C-NMR (CDCl_3_): δ 8.2, 18.7, 19.6, 21.1, 21.2, 25.0, 26.7, 27.5, 33.0, 38.2, 38.3, 42.4, 113.2, 113.5, 122.2, 126.3, 129.5, 135.0, 140.8, 151.5, 153.3; HRMS (ESI+) for C_21_H_31_N_2_O [M+H]^+^: calcd 327.2436; found 367.2439.

#### 3.2.10. Preparation of (5*S*,5'*S*,8*R*,8'*R*)-5,5'-((2*R*,2'*R*,5*E*,5'*E*,7*Z*,7'*Z*)-7,7'-(Ethane-1,2-diylbis(azan-1-yl-1-ylidene))bis(6-methylhept-5-ene-2-yl-7-ylidene))bis(3,8-dimethyl-5,6,7,8-tetrahydronaphthalen-1-ol) (**12a**)

To a solution of compound **6** (91 mg, 0.3 mmol) in dry toluene (1.5 mL), ethyendiamine (10 mL, 0.15 mmol) and anhydrous Na_2_SO_4_ (900 mg) were added. The mixture was maintained at room temperature for 19 h. The solid was filtered, and the solvent was removed under reduced pressured to isolated 92 mg (95%) of the diimine derivative **12a**. IR: 3447, 2925, 1654, 1624, 1458, 1119 cm^−1^. ^1^H-NMR (CDCl_3_): δ 0.96 (3H, d, *J* = 7.6 Hz), 1.18 (3H, d, *J* = 6.8 Hz), 1.20–1.60 (4H, m), 1.76 (3H, s), 1.8–2.10 (6H, m), 2.19 (3H, s), 2.56 (1H, m), 3.07 (1H, m), 3.70 (2H, s), 5.69 (1H, t, *J* = 6.8 Hz), 6.38 (1H, s), 6.51 (1H, s), 7.69 (1H, s); ^13^C-NMR (CDCl_3_) δ 12.4, 19.6, 20.0 (2C), 22.0, 27.5, 27.7, 28.4, 33.3, 39.2, 43.1, 62.1, 114.1, 122.7, 127.5, 135.9, 136.3, 141.5, 144.1, 154.5, 168.7. HRMS (ESI^+^) for C_42_H_61_N_2_O_2_ [M+H]^+^ calcd. 625.4732; found 625.4728.

#### 3.2.11. Preparation of (5*S*,5'*S*,8*R*,8'*R*)-5,5'-((2*R*,2'*R*,5*E*,5'*E*)-7,7'-(Ethane-1,2-diylbis(azanediyl))-bis(6-methylhept-5-ene-7,2-diyl))bis(3,8-dimethyl-5,6,7,8-tetrahydronaphthalen-1-ol) (**12b**)

Diimine **12a** (83 mg, 0.13 mmol) was dissolved in ethanol (2 mL), and a solution of NaBH_4_ (10 mg, 0.26 mmol) in anhydrous ethanol (1 mL) was added. The mixture was maintained for 3 h at 40 °C, cooled, diluted with water (5 mL), treated with a 5% NaOH, and extracted with dichloromethane. The organic layer was washed with brine, dried over K_2_CO_3_, and the solvent was removed under reduced pressure to isolated 79 mg (90%) of ethylendiamine derivative **12b**. IR: 3450, 2925, 1654, 1617, 1578, 1458, 840 cm^−1^.^1^H-NMR (CDCl_3_): δ 0.89 (3H, d, *J* = 6.8 Hz), 1.00–1.25 (4H, m), 1.11 (3H, d, *J* = 6.0 Hz), 1.50–2.0 (8H, m), 1.75 (3H, s), 2.15 (3H, s), 2.50 (1H, m), 2.52 (1H, m), 3.01 (2H, m), 5.09 (1H, br. s), 6.33 (1H, s), 6.48 (1H, s); ^13^C-NMR (CDCl_3_): δ 14.7, 18.8, 19.4, 21.2 (2C), 26.1, 26.6, 26.5, 33.2, 38.2, 42.4, 47.7, 57.3, 113.3, 121.7, 126.8, 127.5, 132.4, 134.8, 140.7, 153.9; HRMS (ESI^+^) for C_42_H_65_N_2_O_2_ [M+H]^+^ calcd. 629.5042; found 629.5038.

#### 3.2.12. Preparation of (*R*,*E*)-6-((1*S*,4*R*)-4,7-Dimethyl-5,8-dioxo-1,2,3,4,5,8-hexahydronaphthalen-1-yl)-2-methylhept-2*-*enoic acid (**13**)

The aldehyde **6** (60 mg, 0.17 mmol) was dissolved in *tert*-butyl alcohol (4 mL) and 2-methyl-2-butene (1 mL). Then, a solution of sodium chlorite (141 mg, 1.56 mmol) and sodium hydrogen phosphate (126 mg, 1.05 mmol) in water (2 mL) was added dropwise over a 10 minute period. The reaction mixture was stirred at room temperature overnight. The solvent was removed under reduced pressured, and the residue dissolved in water (10 mL) and extracted with hexane (2 × 8 mL). The combined organic layers were dried with Na_2_SO_4_, filtered and evaporated under reduced pressure to give 40 mg (72%) of the quinone **13**. IR: 3520, 2930, 2870, 1710, 1690, 1660, 1606, 1516, 1453, 1281, 1128, 890 cm^−1^; ^1^H-NMR (CDCl_3_): δ 0.85 (3H, d, *J* = 6.8 Hz), 1.06 (3H, d, *J* = 6.8 Hz), 1.10–1.30 (2H, m), 1.79 (3H, s), 1.60–2.20 (7H, m), 2.00 (3H, s), 2.78 (1H, m), 2.93 (1H, m), 6.52 (1H, s), 6.80 (1H, t, *J* = 6.8 Hz), 8.20 (1H, br s); ^13^C-NMR (CDCl_3_): δ 11.9, 15.8, 18.5, 19.3, 21.0, 25.2, 26.6, 26.8, 33.4, 35.8, 36.7, 127.1, 133.4, 144.9, 145.0, 145.4, 147.4, 173.2, 187.1, 188.4; HRMS (ESI+) for C_20_H_26_O_4_Na [M+Na]^+^ calcd. 353.1729; found 353.1714.

#### 3.2.13. Preparation of (5*R*,8*S*)-2,5-Dimethyl-8-((*E,R*)-6-methylhept-5-en-2-yl)-5,6,7,8-tetrahydronaphthalene-1,4-dione (**14**)

Leubethanol (74 mg, 0.26 mmol) was dissolved in in *tert*-butyl alcohol (9 mL) and 2-methyl-2-butene (2 mL). Then, a solution of sodium chlorite (216 mg, 3.4 mmol) and sodium hydrogen phosphate (210 mg, 1.8 mmol) in water (2 mL) was added dropwise over a 10 minute period. The reaction mixture was stirred at room temperature overnight. The solvent was removed under reduced pressured, the residue dissolved in 10 mL of water and extracted with hexane (2 × 10 mL). The combined organic layers were dried with Na_2_SO_4_, filtered and evaporated under reduced pressure to give 30 mg of starting material and 35 mg (48%) of the quinone **14**. IR: 2957, 2926, 1718, 1676, 1601, 1540, 1410, 1272, 1201, 1109, 890 cm^−1^; ^1^H-NMR (CDCl_3_): δ 0.78 (3H, d, *J* = 6.8 Hz), 1.01 (3H, d, *J* = 6.8 Hz), 1.10–1.40 (2H, m), 1.49 (3H, s),1.58 (3H, s), 1.60–2.20 (7H, m), 1.95 (3H, s), 2.70 (1H, m), 2.87 (1H, m), 4.93 (1H, t, *J* = 7.2 Hz); 6.44 (1H, s); ^13^C-NMR (CDCl_3_): δ 15.7, 17.6, 18.7, 19.0, 21.1, 25.6, 25.7, 26.3, 26.5, 34.8, 36.6, 37.6, 124.1, 131.5, 133.4, 143.2, 145.5, 147.0, 187.3, 188.4; HRMS (ESI+) for C_20_H_29_O_2_ [M+H]^+^ calcd. 301.2168; found 301.2164.

#### 3.2.14. Preparation of 8-Acetoxyleubethanol (**15**)

Distilled Ac_2_O (0.5 mL) was added to a solution of leubethanol (97 mg, 0.33 mmol) in pyridine (2 mL). After 12 hours the reaction was quenched with ice, one hour later the mixture was extracted with EtOAc and the organic layer was washing with 10% HCl, 5% NaHCO_3_, brine and finally dried over Na_2_SO_4_.The solvent was evaporated under reduced pressure to give a crude oil that was purified by chromatography on silica gel *n*-hexane/EtOAc (95:5) to give 104 mg of acetylated Leub, compound **15** (95%). IR: 2954, 2926, 2870, 1765, 1619, 1571, 1451, 1370, 1207, 1048 cm^−1^. ^1^H-NMR (CDCl_3_): δ 0.99 (3H, d, *J* = 7.1 Hz, 3H), 1.15 (3H, d, *J* = 6.8 Hz), 1.59 (3H, s), 1.68 (3H, s), 2.31 (6H, s), 2.61 (1H, m), 2.95 (1H, m), 5.01 (1H, t, *J* = 7.1 Hz), 6.70 (1H, s), 6.88 (1H, s); ^13^C-NMR (CDCl_3_): δ 17.7, 18.8, 19.3, 21.2 (2C), 21.8, 25.8, 26.3, 27.2, 27.4, 33.2, 38.0, 42.3, 120.3, 124.9, 127.6, 131.2, 131.6, 135.1, 141.2, 148.7, 169.7; HRMS (ESI^+^) for C_22_H_33_O_2_ [M+H]^+^: calcd. 329.2481; found 329.2502.

#### 3.2.15. Preparation of (5*S*,8*R*)-5-((*E,R*)-6-Hydroxy-6-methylhept-4-en-2-yl)-3,8-dimethyl-5,6,7,8-tetrahydronaphthalen-1-yl acetate (**16**)

To a magnetic stirred, ice-cooled solution of diphenyldiselenide (590 mg, 1.89 mmol) in dry dichloromethane (7 mL) was slowly added chilled 33% of hydrogen peroxide (0.21 mL, 64.2 mg, 1.89 mmol). After stirring vigorously for 30 min (white crystals deposit within 10 min), powered anhydrous magnesium sulfate (300 mg) was added and the mixture was stirred for an additional 30 min in the ice-bath. The ice bath was removed, compound **15** (408 mg, 0.86 mmol) was added and the mixture stirred vigorously for 6 h at 25 °C. Chilled 3M *tert*-butyl hydroperoxide (1.4 mL, 4.3 mmol) was added to the reaction mixture which had been immersed in an ice bath; then, after removing the ice bath, the mixture was stirred for 20 h at 25 °C to give a pale orange solution with a lot of white precipitated. The white precipitate (PhSeO_2_H and hydrated MgSO_4_), was filter off and washed with ethyl acetate. The filtrate was concentrated to give an oil. The oil was dissolved in ethyl acetate (100 mL) and washed with 5% Na_2_CO_3_ water, 10% FeSO_4_, water, (sat)NaHCO_3_, water and brine, successively and then, it was dried over Na_2_SO_4_. The solvent was evaporated under reduced pressure to give a crude oil that was purified by chromatography on silica gel *n*-hexane/ethyl acetate (98:2) to give 152 mg of starting material and 245 mg (56%) of allyl alcohol **16**. IR: 3454, 2950, 2922, 2872, 1763, 1618, 1571, 1453, 1370, 1207, 1140, 971, 881 cm^−1^. ^1^H-NMR (CDCl_3_): δ 0.95 (3H, d, *J* = 6.8 Hz), 1.12 (3H, d, *J* = 6.8 Hz), 1.22 (3H, s), 1.23 (3H, s), 2.28 (3H, s), 2.29 (3H, s), 2.62 (1H, m), 2.91 (1H, m), 5.3–5.6 (2H, m), 6.67 (1H, s), 6.87 (1H, s); ^13^C-NMR (CDCl_3_): δ 18.8, 19.3, 21.1, 21.1, 21.6, 27.2, 27.2, 29.7, 29.8, 36.3, 39.3, 41.6, 70.6, 120.4, 126.3, 127.8, 131.6, 135.3, 139.0, 141.1, 148.7, 169.8; HRMS (ESI^+^) for C_22_H_32_O_3_Na [M+Na]^+^: calcd. 367.2249; found 367.2295.

#### 3.2.16. Preparation of (3*R*,7*S*,9*R*,9a*S*)-3,6,9-Trimethyl-7-(2-methylprop-1-enyl)-2,3,7,8,9,9a-hexahydro-1*H*-phenalen-4-yl acetate (**17a**) and (3*R*,7*R*,9*S*,9a*S*)-3,6,9-Trimethyl-7-(2-methylprop-1-enyl)-2,3,7,8,9,9a-hexahydro-1*H*-phenalen-4-yl acetate (**17b**)

A solution of allylic alcohol **16** (70 mg, 0.20 mmol) was cooled to −78 °C and treated dropwise with methanesulfonic acid (65 μL, 1.0 mmol). The solution was warmed to −40 °C and stirred for 12 h, and then triethylamine (0.50 mL) was added. The mixture was warmed to 20 °C, filtered off through a small plug of silica gel with hexane, and concentrate *in vacuo* to afford 63 mg (97%) of a mixture of compounds **17a** + **17b** (2:1).

Compound **17a**. IR: 2925, 2863, 1762, 1598, 1452, 1370, 1209, 1036 cm^−1^. ^1^H-NMR (CDCl_3_): δ 0.86 (3H, d, *J* = 7.1 Hz), 1.19 (3H, d, *J* = 6.9 Hz), 1.67 (3H, s), 1.72 (3H, s), 2.12 (3H, s), 2.29 (3H, s), 2.70 (1H, m), 3.02 (1H, m), 3.65 (1H, m), 5.16 (1H, m), 6.68 (1H, s); ^13^C-NMR (CDCl_3_): δ 16.0, 17.6, 19.9, 21.1, 22.7, 25.3, 27.1, 28.6, 30.8, 31.5, 34.7, 37.3, 39.9, 121.7, 128.8, 131.4, 132.6, 135.0, 135.5, 137.2, 146.9, 169.7; HRMS (ESI^+^) for C_22_H_30_O_2_Na [M+Na]^+^: calcd. 349.2143; found 349.2154.

Compound **17b**. IR: 2925, 2863, 1762, 1598, 1452, 1370, 1209, 1036 cm^−1^. ^1^H-NMR (CDCl_3_) δ 0.72 (3H, d, *J* = 7.1 Hz), 1.20 (3H, d, *J* = 6.9 Hz), 1.68 (3H, s), 1.72 (3H, s), 2.13 (3H, s), 2.29 (3H, s), 2.70 (1H, m), 3.02 (1H, m), 3.63 (1H, m), 3.82 (1H, ddd, *J* = 8.7, 8.7, 8.7 Hz); 5.01 (1H, br d, *J* = 8.7 Hz,), 6.66 (1H, br s); ^13^C-NMR (CDCl_3_): δ 13.6, 17.4, 20.0, 21.1, 23.0, 25.4, 27.2, 28.7, 31.2, 31.4, 33.6, 37.5, 40.3, 121.6, 129.0, 129.8, 131.3, 135.1, 135.2, 138.1, 149.6, 169.7.

#### 3.2.17. Preparation of (3*R*,7*S*,9*R*,9a*S*)-3,6,9-Trimethyl-7-(2-methylprop-1-enyl)-2,3,7,8,9,9a-hexahydro-1*H*-phenalen-4-ol (**18**)

Acetyl compound **17a** (53 mg, 0.16 mmol) was dissolved in KOH/MeOH (5 mL, 2M) and stirred for 12 h. After that time, the solvent was evaporated, the residue was dissolved in water and extracted with ethyl acetate. The organic layer was dried over Na_2_SO_4_, and the solvent was evaporated under reduced pressure to give a crude oil that was purified by chromatography on silica gel *n*-hexane/ ethyl acetate (98:2) to give 35 mg 63%) of alcohol compound **18**. IR: 3516, 2958, 2921, 2866, 1589, 1450, 1412, 1377, 1237, 1177, 1031, 908, 732 cm^−1^. ^1^H-NMR (CDCl_3_): δ 0.70 (3H, d, *J* = 7.0 Hz); 1.28 (3H, d, *J* = 6.8 Hz); 1.67 (3H, s); 1.71 (3H, s); 2.10 (3H, s); 2.68 (1H, m); 3.11 (1H, m); 3.61 (1H, q, *J* = 9.0 Hz); 4.98 (1H, dt, *J* = 9.3, 1.3 Hz); 6.42 (1H, s); ^13^C-NMR (CDCl_3_): δ 13.3, 17.4, 19.9, 22.8, 27.8, 28.5, 29.4, 31.4, 32.0, 33.2, 37.7, 40.8, 115.4, 125.7, 128.4, 129.6, 130.6, 135.1, 138.8, 151.4. HRMS (ESI^+^) for C_20_H_29_O [M+H]^+^: calcd. 385.2218; found 385.2255.

### 3.3. In Vitro Antimycobacterial Evaluation 

The antimycobacterial activity was assessed against *M. tuberculosis* H37Rv ATTC 27294 susceptible to all five first-line anti-TB drugs (streptomycin, isoniazid, rifampin, ethambutol, and pyrazinamide) in a modified Microplate Assay Blue Alamar [22,23]. The compounds for *M. tuberculosis* bioassays were prepared at a concentration of 1 mg/mL in 2.5% DMSO in Middlebrook 7H9 (Becton Dickinson and Co., Sparks, MD, USA) broth. All solutions were sterilized by filtration using 13 mm diameter PTFE acro-discs (0.22 μm pore size, Millipore Co., Bedford, MA, USA). The concentrations for organic compounds used ranged from 100 μg/mL to 0.78 μg/mL, results are reported as minimal inhibition concentration (MIC). Ethambutol (EMB) was used as positive control. All biological assays were developed at least by triplicate.

### 3.4. Cytotoxicity Assay 

Cytotoxicity was determined according to the MTT method [24] using African green monkey kidney epithelial cells (Vero cells) grown in RPMI-1640 medium supplemented with penicillin (100 units/mL), streptomycin (100 μg/mL), and fetal bovine serum (10%) at 37 °C under 5% CO_2_. In the confluence, 4000 cells per well was applied to a 96-well microtitre plate. After incubation for 24 h (37 °C, 5% CO_2_), 10 μL of a solution containing concentrations ranging from 500 to 0.5 μg/mL of the compound under evaluation was added and incubated for 48 h. 25 μL of MTT (4 mg/mL in PBS) was added to each well and incubated for another 3 h. The solution in each well was removed and DMSO (200 μL) was then added to each well. The absorbance was recorded on a microplate reader at a wavelength of 575 nm. The CC_50_ value was calculated by linear regression as the concentration of the compound inhibiting 50% cellular viability.

## 4. Conclusions 

The results found in this study regarding the antimicrobial activity indicate that there are few possibilities of modification on the leubethanol molecule. The dimer of leubethanol, compound **12b** showed a higher selectivity index than the natural compound.
